# Flat Wall Proximity Effect on Micro-Particle Sedimentation in Non-Newtonian Fluids

**DOI:** 10.1038/s41598-020-59386-5

**Published:** 2020-02-17

**Authors:** Vahideh Farzam Rad, Ali-Reza Moradi

**Affiliations:** 1 Department of Physics, Institute for Advanced Studies in Basic Sciences, PO Box 45195-1159 Zanjan, Iran; 2 School of Nano Science, Institute for Research in Fundamental Sciences (IPM), PO Box 19395-5531, Tehran, 19395 Iran

**Keywords:** Fluid dynamics, Imaging techniques, Applied optics, Optical techniques

## Abstract

We investigate the sedimentation of colloidal micro-spheres and red blood cells (RBCs) in non-Newtonian fluid - silicone oil with different viscosities. We use digital holographic microscopy (DHM) to obtain volumetric information of the sedimenting micro-objects. Especially, the numerical refocusing feature of DHM is used to extract the depth information of multiple particles moving inside the fluid. The effects of proximity to a flat wall and the non-Newtonian behavior on the sedimenting micro-spheres and RBCs are studied by trajectory analyzing and velocimetry. We observe that for lower viscosity values the proximity effect is more pronounced. The variation rate of the particle falling velocities versus their distance to the flat wall decreases by increasing the viscosity of the fluid. For RBCs, however, the decreasing of the velocity variations have a smoother trend. The experimental results verify the theoretical prediction that, similar to Newtonian case, a correction factor in Stokes’ law suffices for describing the wall effect.

## Introduction

Several phenomena, in different scales, in biology, geology, and industry include settling of suspended particles in fluids. Understanding the behavior of sedimenting particles in various circumstances is important in fundamental theoretical studies as well as in applications such as separation in multiphase systems, membrane transport, hydraulic transport, gravity-driven drainage of paints, coating of thin films, and the motion of drilling muds^1–6^. In the investigation of the sedimentation behavior processes and parameters such as the gravitational force, the hydrodynamic interactions, the Brownian motion of the particles, the inherent motility of the particles, and the influences of the boundaries have to be considered. The proximity of the particles to the boundaries, for example a flat wall, may affect the overall hydrodynamic interactions and the resulting motion of the suspended particles sedimenting against the gravity in the vicinity of the wall or suspending in a linear shear flow^7–9^. The level of the influence, obviously, depends on the geometry and morphology of the wall, size of the particles, and ratio of the particle size to its distance from the wall^10,11^. The influence of walls in containers with cylindrical geometry on the hydrodynamic behavior of rigid particles has been investigated in various directions; For example, Charru *et al*. studied the behavior of the particles near the bottom boundary of the container^10^, and Eisenberg *et al*. investigated the particles motion close to the wall when a linear shear flow applied to the fluid and also considered the effect of the roughness of the bottom boundary, which causes resuspension of the settled particles^12^. However, the earliest work on modeling of hydrodynamic behavior of the particles in motion near to a flat wall was done by Goldman *et al*.^13,14^. On the one hand, most of the experimental investigations on hydrodynamics of the sedimentation process have been performed in the macro-scales and in the field of geology. On the other hand, understanding particle-wall interactions in microscopic scales is of high practical significance in complex fluids and advanced materials technologies^15,16^. Recently, we studied the proximity effect of a flat wall on sedimentation rate of microscopic colloidal particles in a Newtonian fluid^17^. In order to ignore the influence of particle-particle hydrodynamic interaction a dilute suspension of particles was used.

The aforementioned researches have been focused on particles settling in viscous Newtonian fluids and little is known about the effect on micro-particles sedimenting in non-Newtonian shear-thinning fluids. In this report, we address the proximity effect on sedimenting micro-objects in non-Newtonian fluids. We use a systematic study based on the use of single exposure digital holographic microscopy (DHM) to extract the full morphometric information of collection of falling colloidal micro-spheres and red blood cells (RBCs). Therewith, the influence of the particle rigidity and shape on the behavior may be examined. Three-dimensional (3D) imaging through DHM is very useful for visualizing the distributed micro-particles in a volume of a fluid. Most of the 3D imaging methodologies for detection and tracking of collective behavior of micro-objects, for example confocal microscopy, are based on scanning and require electro-mechanical adjustment to image the particles away from the focal plane. Flow measurement methodologies such as astigmatism particle tracking velocimetry works only for spherical shape particles and cannot provide 3D image of the moving micro-objects, and also their measurement region is limited due to the calibration procedure^18–20^. DHM in transmission mode, however, provides an elegant solution for these technical concerns. DHM is based on the combination of conventional microscopy and laser interferometry. The recorded interference pattern between the light scattered from the specimen and a reference wave is called a digital hologram^21^. The recorded digital holograms are subjected to numerical reconstruction process and give the whole-field information about the sample under investigation. DHM in general is an effective method for non-destructive and quantitative visualization of “phase” objects, such as living cells^22,23^. It provides real-time 3D imaging data at time scales from milliseconds to hours. Therefore, it is a suitable approach to study dynamic phenomena, such as the ones within microfluidics^24–27^. Besides, a feature that may be taken into use in studying of volumetric or multiple micro-objects is numerical refocusing; it enables us to focus on the different layers of the specimen under study through the simple numerical propagation of the recorded holograms^17,28–30^.

## Theory

According to Stokes’ law, in low Reynolds numbers (*Re*s), a hard sphere of radius $a$ moving with a velocity of ${\upsilon }_{0}$ in an unbounded quiescent fluid of viscosity $\eta$ will experience a hydrodynamic drag force opposite to its direction of motion that is expressed by:

1 $${F}_{0}=-\,6\pi \eta a{\upsilon }_{0}.$$

In the case of particle sedimentation the gravitational force cause the particles to move downward and the terminal velocity of their sedimentation can be governed by Eq. (1); The drag force is replaced by Δ$\rho Vg$, where Δ$\rho$ is the difference between the densities of the particle and the surrounding medium, $V$ is the volume of the particle, and $g$ is the gravitational acceleration constant. For particles larger than a few micrometers, the gravitational force dominates over the diffusive force, and the motion is considered only in gravitation direction. It is known that sedimenting velocity is hindered when the particle is close to a boundary, due to increase of the drag force^8,31,32^. For the motion of micro-spheres near a flat wall in Newtonian fluids we have shown that the effect of a flat wall may be represented by multiplying a correction factor (${\lambda }_{h}^{-1}$) to the drag force, which was in agreement with the empirical results^13,17^. ${\lambda }_{h}^{-1}$ is a power series of the ratio of the radius of a sedimenting micro-sphere ($a$) and its distance from the flat wall ($h$), and it is independent of the Reynolds number:

2 $${\lambda }_{h}^{-1}\cong 1-\frac{9}{16}(\frac{a}{h})+\frac{1}{8}{(\frac{a}{h})}^{3}-\frac{45}{256}{(\frac{a}{h})}^{4}-\frac{1}{16}{(\frac{a}{h})}^{5}+O{(\frac{a}{h})}^{6}.$$

The flat wall proximity effect on the increase of drag force was effective for distances up to several particle sizes. In larger *h*/*a*, ${\lambda }_{h}\approx 1$ and Stokes’ law is valid, however, in very small distances between the particle and the wall (of the order of the particle size), the drag force increases dramatically. The correction factor for a sphere may be obtained by “the method of reflection”, in which, the several particle-boundary interactions are represented by various high order terms whose combination results in the the velocity and the pressure fields expressions^33^. The analytical solution even in low Reynolds numbers is difficult to proceed^7,13,34^. Researches on particles sedimentation have been performed mostly for Newtonian fluid case. Here, we address the wall effects on micro-particles settling in non-Newtonian shear-thinning fluids. Non-Newtonian fluids possess specific behavior on characteristics such as plasticity and yield stress. A number of models to explain the relationship between shear stress and shear rate of non-Newtonian fluids are available^35^. The model with least complexity is the so-called power-law model. The wall effects on a sphere settling in power-law fluids have been investigated by the use of finite-element and finite-volume methods^36^. It is shown that the terminal sedimenting velocity for a shear-thinning viscoelastic fluid confined in a cylinder is influenced by shear-dependent viscosity, but further the wall effects are eliminated by the fluid elasticity^37^.

For a Newtonian fluid the drag coefficient, *C*_*D*_, and *Re* may be expressed by:

3 $${C}_{D}=\frac{8}{3}\frac{\Delta \rho ag}{{\rho }_{f}{v}_{\infty }^{2}},\,{Re}=\frac{2{\rho }_{f}{v}_{\infty }a}{\eta },$$

where, ${\rho }_{f}$ is the fluid density and ${v}_{\infty }$ is the settling velocity of the micro-sphere in an unbounded fluid. At low *Re*s the *C*_*D*_ and ${v}_{\infty }$ expressions are simplified to:

4 $${C}_{D}=\frac{24}{{Re}},\,{v}_{\infty }=\frac{2\Delta \rho {a}^{2}g}{9\eta }.$$

In order to quantify the retarding effect of the wall on the settling particles, the wall factor $\frac{{v}_{h}}{{v}_{\infty }}$ is defined in which, ${v}_{h}$ is the micro-particle terminal settling velocity in a bounded fluid media at a distance *h*. For a Newtonian fluid the wall factor is a function of the particle size and *h*^17^. It can be shown, by dimensional arguments, that also for a non-Newtonian fluid at low *Re*s the relation between the drag coefficient and the *Re*_NN_ (*Re* for a power-law fluid) has the same form, but has an additional correction factor^38^:

5 $${C}_{D}=\chi (n)\frac{24}{R{e}_{{\rm{NN}}}},$$

where:

6 $$\chi (n)=-\,1.1492{n}^{2}+0.8734n+1.2778$$

is obtained by fitting the numerical solution and *n* is the power-law index. The value of wall factor in non-Newtonian case is a function of *n*, *Re*, $\frac{a}{h}$ and $\eta$ for the sedimenting particle. The effect of *n* is embodied in *Re*_NN_. Therefore, also for non-Newtonian fluid the drag force in the presence of the flat wall is similar to the one of unbounded case, but with a correction factor that is a function of not only the $\frac{a}{h}$ parameter, but also depends on the parameters related to power-law behavior.

## Results and Discussion

**Figure 1:**
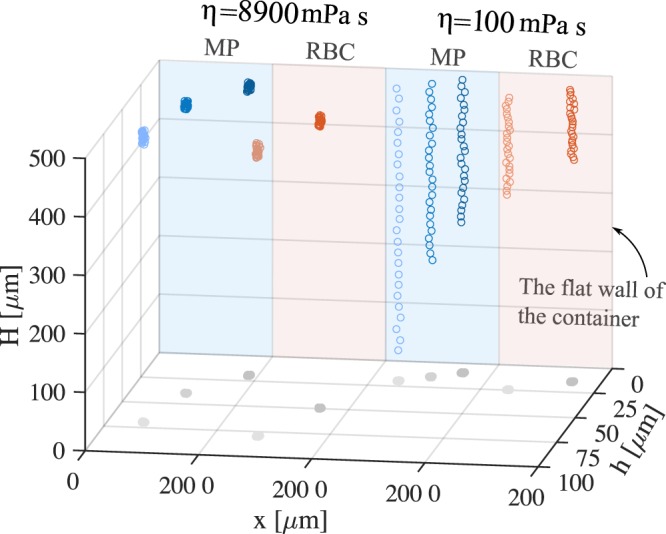
3D trajectories of several micro-particles (MPs) and RBCs, in different distances from the flat wall of the container within 5 s and in two different viscosities (100 mPa s and 8900 mPa s). The bluish circles show the sedimenting polystyrene micro-particles positions; lighter colors indicate farther ones. Similarly, the reddish circles show the RBCs trajectories.

The numerical refocusing feature of DHM is used in this research to extract the depth information of multiple particles falling inside the fluid. The methodology is described in Methods section. Figure 1 shows the 3D trajectories of several sedimenting micro-particles and RBCs for the first 5 s of their appearance in the camera’s field of view. Lighter colors correspond to higher distances of the micro-objects from the flat wall. The trajectories have been obtained by analyzing the recorded holograms and having the information of data acquisition parameters. We have shown the results for the lowest (100 mPa s) and highest (8900 mPa s) examined viscosities of silicone oil. The center of mass of the reconstructed images have been considered for the position of the sedimenting polystyrene micro-particles (bluish circles) and RBCs (reddish circles). Each data point is obtained from an ensemble of micro-particles and RBCs. The size of the ensemble depends on the available micro-objects at similar distances from the wall. Several points may be visually concluded from the trajectories: The sedimentation happens almost in the direction of gravitational force, which indicates that the diffusive force can be neglected comparing with the gravitational force. This can be confirmed from the superimposed trace shadows of the micro-objects on the bottom surface of Fig. 1. The micro-particles and the RBCs falling at distances below few times of their sizes feel the presence of the wall as their settling velocities are hindered and they travel less trajectory within the same duration. The effect is more pronounced for the lower viscosities. The effect is also more visible for micro-particles than RBCs. However, RBCs generally travel slower than the micro-particles which can be attributed to their shapes, densities and permeabilities.

**Figure 2:**
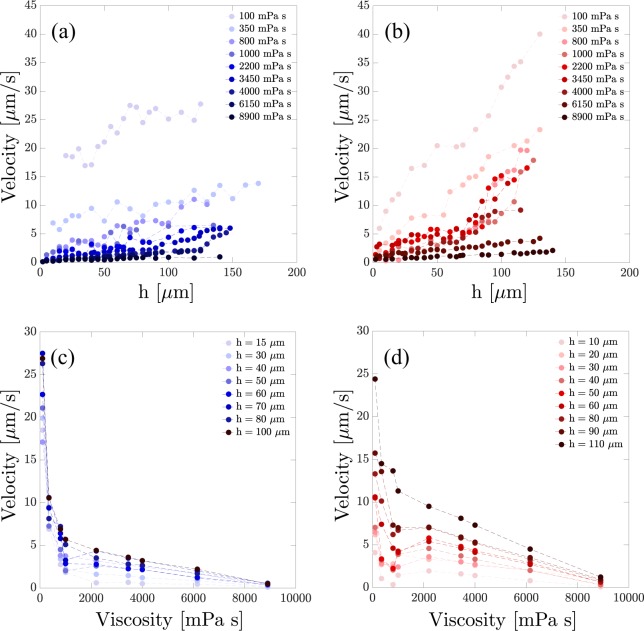
The velocity of the sedimenting (**a**) micro-particles and (**b**) RBCs versus their distances from the flat wall of the container for various viscosities. Lighter colors correspond to the lower viscosities. (**c**,**d**) Velocity vs. viscosity curves for micro-particles and RBCs, respectively, at various distances from the flat wall.

**Figure 3:**
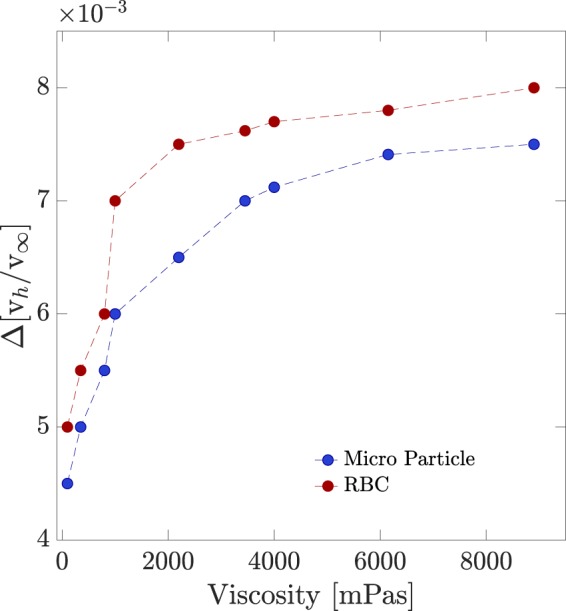
Slope of wall factor variations, Δ$[\frac{{v}_{h}}{{v}_{\infty }}]$, of sedimenting micro-particles and RBCs vs. their distances from the flat wall. In Supplementary 1 the height changes as a function of time for various viscosities are shown.

For more quantitative assessment of the proximity effect we have measured the variations of the settling velocities for several micro-particles and RBCs falling at different distances from the flat wall. The procedure was applied for all the viscosities. The results are presented in Fig. 2. Figure 2(a,b) correspond to the cases of micro-particles and RBCs, respectively. The results show that, overall, the sedimenting velocity is lower for higher viscosities. Further, the velocity variations in high viscosities is less. The measurements show that RBCs settle down slower than micro-particles which also was visible from their trajectories (Fig. 1). The slope of velocity variations vs. distance for micro-particles changes smoother than for RBCs. In order to verify that a correction factor, similar to the case of Newtonian fluids may suffice for describing the wall effect, we have conducted investigations on the velocity vs. viscosity trends. Figure 2(c,d) show the similar trend for all the viscosities including the ones in the non-Newtonian range for micro-particles and RBCs, respectively. This, indeed, is the verification of our theoretical prediction. We have presented the variations of the wall factor for all the viscosities in Fig. 3. For micro-particles and RBCs two categories are distinguishable. In the first one (viscosities below 2000 mPa s) the wall factor varies dramatically while in the other one the changes are smoothly. In the lower viscosities the deviation of the drag force from the unbounded drag force is substantially increased. Knowing that non-Newtonian behavior appears in higher viscosities, the results show that, generally, the proximity effect is less pronounced for non-Newtonian fluids. Therefore, we show that, in microscopic ranges, the shear-thinning effect detracts the flat wall retardation effect on micro-spheres and RBCs settling in shear thinning fluids. The impact of the shear-thinning effect is more pronounced for micro-spheres than RBCs in spite of their higher weights; it means that with increasing the viscosity the falling micro-particles reach to their final velocity earlier, and this is attributed to the morphology and structure of the cells.

In conclusion, we measured the proximity effect of a flat wall on the sedimentation of colloidal micro-spheres and RBCs in non-Newtonian fluids by the use of digital holographic microscopy. DHM provides quantitative phase contrast and 3D imaging in arbitrary time scales that makes it a suitable method to investigate various phenomena including the dynamic behavior of colloids. We have observed that for lower viscosity values the proximity effect is more pronounced. The variation rate of the particles falling velocities versus their distance to the flat wall decreases by increasing the viscosity of the fluid. For RBCs, however, the decreasing of the velocity variations have a smoother trend. The sedimenting phenomenon is ubiquitous in nature and most of them include also the presence of boundaries, for example the motion of biological micro-swimmers near boundaries^39^ and motion of colloidal particles in micro-fluidic channels^2^. The results of this research might have usefulness in such studies; particularly, for the flow of RBCs in vessel and blood clotting as blood has non-Newtonian fluidic behavior^40^. The presented methodology is also capable of being used for different applications in study of colloidal dynamics where 3D information in real time is required.

## Methods

### Sample preparation

Polystyrene micro-spheres of 20 ± 1 *μ*m diameter (2*a*) and red blood cells, which typically have diameters of 6–8 *μ*m, were used as sample. They were dispersed in silicone oils with viscosities of 100, 350, 800, 1000, 2200, 3450, 4000, 6150, and 8900 mPa s. It is known that at higher viscosities silicone oil shows non-Newtonian behavior^41^. The density of the micro-particles which were dispersed in the chamber was chosen in the way that, on the one hand, several micro-particles at different distances from the chamber wall can be observable within the field of view of the imaging system, and, on the other hand, the hydrodynamic interactions between the micro-particles can be neglected.

For the RBC experiments, freshly collected human blood, drawn from clinically healthy donors, was obtained from the Blood Bank of Zanjan, Iran. The plasma and buffy coat of fresh blood were separated by centrifugation at 3000 g for 10 min at 4 °C temperature, and the separated layers were removed by careful aspiration. Then, cells were resuspended in physiological solution (NaCL, 150 mM). The cells were then washed three more times with the same buffer, and diluted to obtain a 0.1% hematocrit value, which is the suitable concentration for microscopic experiments that require a single-cell or few cells in the field of view. The RBC specimens were kept in water bath at 37 °C before the experiments. In each experiment, a droplet of the suspension was added carefully to the upper surface of the silicone oil container, at a close distance (approximately 1 mm) to the inner flat wall, and live monitored. We recorded the holograms at the speed of 10 fps. The experimental protocols were conducted in accordance with the regulations and policies of the Zanjan Blood Bank.

### Experimental setup

**Figure 4:**
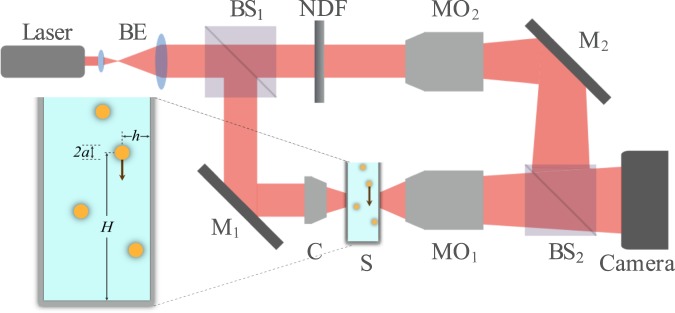
Scheme of the experimental setup; BE: beam expander, BS: beam splitter, NDF: neutral density filter, MO: microscope objective, M: mirror, S: sample, C: condenser.

Figure 4 shows the experimental setup, which is a Mach-Zehnder based transmission mode DHM. The laser beam (MEOS, 632.8 nm, 5 mW) is expanded by the beam expander (BE) and split into two identical beams by a 50:50 beam splitter (BS_1_). One of the beams is directed onto the sample (S) through mirror M_1_ and the condenser (C). The transmitted beam from the sample that contains the information of it is then collected by a 40× microscope objective (MO_1_, Olympus, NA = 0.65, WD = 0.17 mm) and is sent through BS_2_ onto a digital camera (DCC1545M, Thorlabs, 8-bit dynamic range, 5.2 *μ*m pixel pitch). The transmitted beam from the beam splitter BS_1_, is the reference beam and is sent onto the camera, to interfere with the object beam, through the mirror M_2_ and the beam splitter BS_2_. A slight angle applied between the reference and object beams ensures of off-axis DHM arrangement. The successive recorded interference patterns are call holograms and will be subjected to numerical reconstruction process. The microscope objective MO_2_ is similar to MO_1_ and is used to adjust the curvature of the interfering beams, and the neutral density filter (NDF) is used to match their intensities to obtain fringes with maximum contrast. A successful numerical reconstruction process requires uniform, linear and high-contrast fringes. The sample chamber was a 3.5 ml quartz cuvette cell with 10 mm × 10 mm cross-section (Azzota) and was positioned before MO_1_. Its enlarged scheme is shown in the inset of Fig. 1; *H* and *h* are the distances of the sedimenting micro-object from the bottom surface and the inner side of the flat wall of the chamber, respectively. The distances of the settling micro-objects from the side walls and the bottom surface are much beyond their distances from the flat wall to guarantee that only the front flat wall effect is considered.

### Numerical processing

When the laser beam is transmitted through an object its phase and amplitude are changed. The spatial part of the transmitted light is expressed as $\overrightarrow{E}(x,y)={\overrightarrow{E}}_{0}(x,y)\,\exp \,(i\phi (x,y))$, where ${E}_{0}(x,y)$ and $\phi (x,y)$ are the amplitude and the phase, respectively. An object is called “phase object” if the changes in the amplitude is negligible and only phase of the light undergoes appreciate changes. However, bright field microscopies, even qualitatively, are not able to see phase objects because image detectors such as digital cameras, photographic plates, and human eye can detect only the intensity of the light-wave. Therefore, the 3D information about the object that is included only in the phase will be lost. Holography is the elegant method to preserve and detect the phase variations; in holography the sample beam is superposed with a definite reference beam before the detector^42^. Conventional holography has several drawbacks including the requirement to the photographic development and extra facilities for reconstruction, and more importantly the limitations to study dynamic samples. Digital holography, i.e. recording the interference patterns on digital sensors and reconstructing them numerically, is the solution to overcome these shortcomings^43,44^. The interference pattern of the beams can be expressed as:

7 $$\begin{array}{rcl}I(x,y) & = & |{\overrightarrow{E}}_{0s}(x,y){|}^{2}+|{\overrightarrow{E}}_{0r}(x,y){|}^{2}\\  &  & +\,2\{{\overrightarrow{E}}_{0s}(x,y).{\overrightarrow{E}}_{0r}(x,y)\\  &  & \times \,\cos \,[{\phi }_{s}(x,y)-{\phi }_{r}(x,y)]\},\end{array}$$

where ${E}_{0s}$ and ${\phi }_{s}$, and ${E}_{0r}$ and ${\phi }_{r}$ are the amplitudes and phases of the sample and the reference beams, respectively. The phase difference, ${\phi }_{s}-{\phi }_{r}$, is proportional to the optical path length difference of the two beams and can be used to measure the thickness of the transparent sample at any point $(x,y)$, if the illumination is monochromatic and the variations in the refractive index of the sample are neglected.

3D trajectory of sedimenting micro-spheres and RBCs may be obtained through numerical reconstruction of the recorded holograms. The velocity of them can also be obtained by knowing the recording frame rate and the time of sedimentation. The reconstruction process includes simulating the illumination of the recorded holograms by the reference laser beam followed by a diffraction into the plane where the image is planned to form. Angular spectrum propagation approach in scalar diffraction theory is shown to be a suitable method to perform the aforementioned processes^45^. In the reconstruction process, either conventionally or digitally, two terms of the recorded interference pattern after illumination by the reference beam, include the whole information of the sample and will lead to a virtual image and a real image. The other terms are the zero-order terms that cause noises in the final reconstructed images and have to be removed within the reconstruction process^46^.

The angular spectrum i.e. the Fourier transform of the light-wave at the hologramÕs plane, ${E}_{s}(x,y,z=0)$, that is obtained through numerical illuminating of the recorded digital hologram by the reference wave is:

8 $$\begin{array}{rcl}{\rm{FT}}\{{E}_{s}(x,y)\} & = & {\tilde{E}}_{s}(u,v)\\  & \equiv  & \int {\int }_{-\infty }^{\infty }\,{E}_{s}(x,y,0){e}^{-2\pi i(ux+vy)}dxdy,\end{array}$$

where *u* and *v* are the spatial frequencies in *x* and *y* directions, respectively. In the Fourier domain, the zero term and the conjugate real image are filtered out, and through an inverse Fourier transform, the modified light-wave is obtained as:

9 $$\begin{array}{rcl}{E}_{s}^{F}(x,y,0) & = & {{\rm{FT}}}^{-1}\{{\tilde{E}}_{s}^{F}(u,v,0)\}\\  & \equiv  & \int {\int }_{-\infty }^{\infty }\,{\tilde{E}}_{s}^{F}(u,v,0){e}^{2\pi i(ux+vy)}dudv,\end{array}$$

where ${\tilde{E}}_{s}^{F}$ is the filtered angular spectrum of *E*_*s*_. Complex amplitude at an arbitrary plane located at $z=d$ is obtained by free space propagating of ${E}_{s}(x,y,0)$ to a distance *d*:

10 $$\begin{array}{rcl}{E}_{s}^{F}(x,y,d) & = & \int {\int }_{-\infty }^{\infty }\,{\tilde{E}}_{s}^{F}(u,v,0){e}^{ikd\sqrt{1-{\lambda }^{2}{u}^{2}-{\lambda }^{2}{v}^{2}}}\\  &  & \times \,{e}^{2\pi i(ux+vy)}dudv.\end{array}$$

*λ* is the wavelength of the laser beam. The whole process can be summarized by the following expression:

11 $${E}_{s}^{F}(x,y,d)={{\rm{FT}}}^{-1}\{{[{\rm{FT}}\{{E}_{s}(x,y,0)\}]}^{F}{e}^{ikd\sqrt{1-{\lambda }^{2}{u}^{2}-{\lambda }^{2}{v}^{2}}}\}.$$

${\phi }_{s}$, the phase of the object, and *I*_*s*_, the intensity of the object, are calculated from the complex amplitude as:

12 $${\phi }_{s}(x,y,z)=\arctan \frac{\Im [{E}_{s}^{F}(x,y,z)]}{\Re [{E}_{s}^{F}(x,y,z)]},$$

13 $${I}_{s}(x,y,z)=|{E}_{s}^{F}(x,y,z){|}^{2}.$$

**Figure 5:**
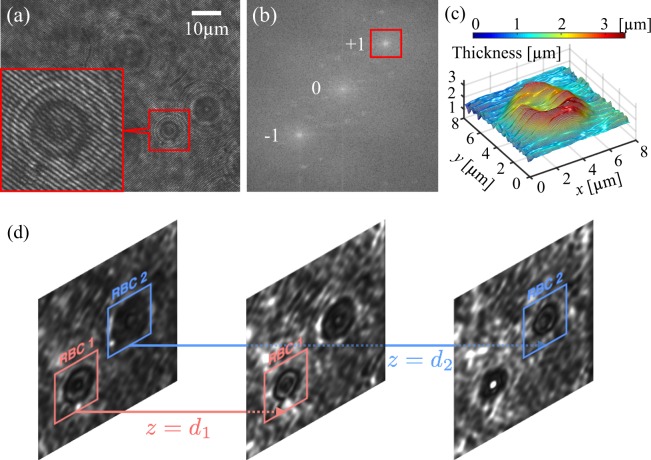
Numerical reconstruction; (**a**) Hologram of a sedimenting RBC. A closer view of the hologram is also shown; (**b**) Fourier spectrum of the hologram. +1 spot is chosen for numerical propagation in the Fourier domain; (**c**) Reconstructed 3D image of the RBC; (**d**) Numerical re-focusing of two RBCs is different distances from the flat wall. In Supplementary Information 2 a movie of the digital holograms of micro-particles sedimentation is shown.

If needed a smoothing process on the obtained phase and amplitude can be performed within the numerical reconstruction process. Equation (12) gives the phase-map of the object, which embeds the 3D information of phase objects such as RBCs. The phase map is proportional to the optical path length, $\phi =\frac{2\pi }{\lambda }nL$, where *n* is the refractive index of the medium and *L* is the physical length that the light beam propagates, e.g. the thickness of RBCs. The phase changes when either the shape or the refractive index of the object under study is changed. Assuming negligible changes of the refractive index of the object the phase distribution directly leads to the surface 3D profile of it. The phase obtained from Eq. (12) is in the range of $[-\frac{\Pi }{2},\frac{\Pi }{2}]$, which makes discontinuities throughout the phase map. The discontinued phases are converted to continuous phase maps by the unwrapping process. We have used Goldstein’s branch-cut unwrapping algorithm in this paper^47^. Figure 5(a) shows a typical recorded hologram of a sedimenting RBC. The interferometric fringes are visible in the enlarged pattern. In the Fourier domain the frequencies associated with either virtual or real images are selected from the Fourier spectrum, shifted into the center of the domain and subjected to numerical propagation e.g. “+1” term in Fig. 5(b). In DHM, in order to remove the effects of the background contaminations and the aberrations of the optical train from the extracted data, acquisition of a reference hologram is required. Within the reconstruction process its phase is subtracted from the ones for the sample holograms. In this research the reference hologram for each viscosity of silicone oil was recorded separately in the beginning of the experiment when the chamber was fully filled with the oil. Figure 5(c) illustrates the final 3D image of the RBC resulted from the numerical reconstruction.

The intensity image, similar to the bright-field microscopy image, can be obtained through Eq. (13). However, DHM, additionally, provides numerical focusing. Therefore, instead of mechanical adjusting of the objective position in conventional microscopes, by changing the propagation distance, *d*, in Eq. (11), the complex amplitude may be computed at any desired axial plane and the corresponding intensity image may be formed. This feature of DHM is used in this research in order to measure the axial distance of the sedimenting micro-objects from the flat wall. For quantitative assessment of the axial distance the hologram is reconstructed with a *d* so that the object is seen in its sharpest form in the intensity or the 3D image. Figure 5(d) shows the reconstructed intensity image of the two RBCs falling in different axial distances that are shown in the hologram of Fig. 5(a). By numerical refocusing the distances *d*_1_ and *d*_2_, at which, one of the two RBCs has a sharp image (as a fingerprint of its position) are measured. The process is performed for several RBCs and polystyrene micro-particles and the axial distances of them from the flat wall are determined. The lateral position of them can also be measured easily, by having the pixel size of the camera and the field of view seen by the camera. Hence, by DHM experiments the 3D trajectory and the falling velocity of multiple micro-objects at different axial distances will be known and will be used to examine the influence of the proximity to the flat wall.

## Supplementary information

Supplementary Information.

Supplementary Video.
