# Supplementary material for: Flat Wall Proximity Effect on Micro-Particle Sedimentation in Non-Newtonian Fluids
Source: Sci Rep. 2020 Feb 17;10:2741. doi: 10.1038/s41598-020-59386-5 (PMC7026440; doi:10.1038/s41598-020-59386-5)
Supplement: Supplementary file 1 — Supplementary Information. [file 41598_2020_59386_MOESM1_ESM.pdf]

Supplementary Information

Flat Wall Proximity Effect on Micro-Particle Sedimentation in Non-Newtonian Fluids

Vahideh Farzam Rad[1], Ali-Reza Moradi[1,2,*]

[1] Department of Physics, Institute for Advanced Studies in Basic Sciences, PO Box 45195-1159, Zanjan, Iran
[2] School of Nano Science, Institute for Research in Fundamental Sciences (IPM), PO Box 19395-5531, Tehran, 19395, Iran

*Corresponding author: *moradika@iasbs.ac.ir*

Contents:

Supplementary Figure S1:

The height variations for various viscosities and distances to the flat wall.

Supplementary Video S1:

Holography of micro-particles sedimentation at different distances to the flat wall.

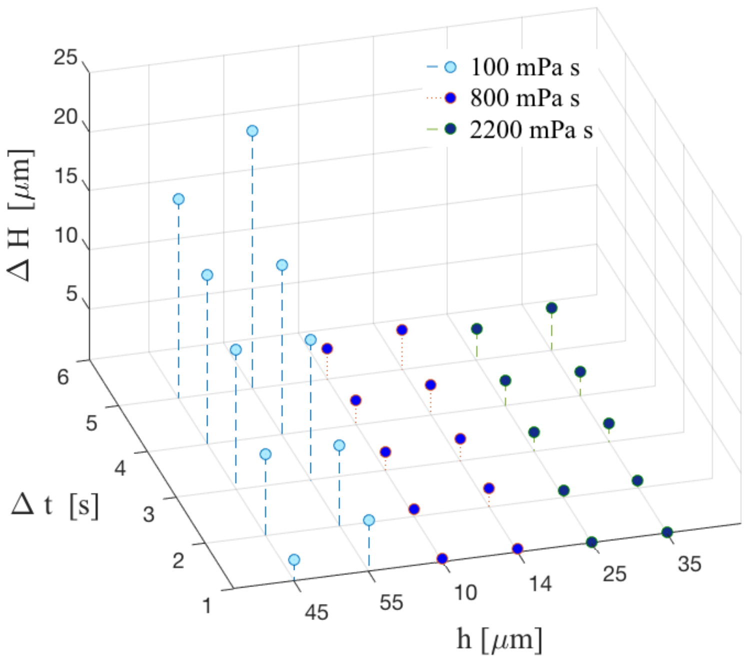

Figure S-1: The height changes of sedimenting micro-particles in different Δt =1 s time durations after recording the holograms for various viscosities and distances to the flat wall.

The figure is the height changes of the sedimenting polystyrene micro-particles at each $\Delta t = 1$ s from the appearance of the particles in the camera's field of view, for three different viscosities (100, 800, and 2200 mPa s) and for two different distances to the wall. The particles sedimenting in a fluid experiences gravity and drag forces. According to the dependence of density to viscosity, the drag force in the liquid with higher viscosity is increased and leads to lower sedimenting velocities, which is in agreement with these results. The height changes as a function of time are increased for all the viscosities. In Fig. 3 of the paper, the effect of the proximity to the flat wall for various viscosities is shown in details.

Supplementary Video S1:

Holography of micro-particles sedimentation at different distances to the flat wall; The movie is showing the sedimentation of polystyrene micro-particles at different distances to the flat wall in a liquid with viscosity $\eta = 800$ mPa s. The almost vertical trajectory of the sedimenting micro-particles, is evident as also indicated by the data shown in Fig. 1 of the paper.
